# The Impact of Maternal Anxiety on Early Child Development During the COVID-19 Pandemic

**DOI:** 10.3389/fpsyg.2021.792053

**Published:** 2021-12-22

**Authors:** Ljiljana Jeličić, Mirjana Sovilj, Ivana Bogavac, And̄ela Drobnjak, Olga Gouni, Maria Kazmierczak, Miško Subotić

**Affiliations:** ^1^Cognitive Neuroscience Department, Research and Development Institute “Life Activities Advancement Center,” Belgrade, Serbia; ^2^Department of Speech, Language and Hearing Sciences, Institute for Experimental Phonetics and Speech Pathology, Belgrade, Serbia; ^3^Cosmoanelixis, Prenatal & Life Sciences, Athens, Greece; ^4^Prenatal Sciences Research Institute, Athens, Greece; ^5^Institute of Psychology, University of Gdansk, Gdansk, Poland

**Keywords:** perinatal mental health, maternal anxiety, infant development, social support, COVID-19 pandemic, COVID-19-related fear

## Abstract

**Background:** Maternal prenatal anxiety is among important public health issues as it may affect child development. However, there are not enough studies to examine the impact of a mother's anxiety on the child's early development, especially up to 1 year.

**Objective:** The present prospective cohort study aimed to examine whether maternal trait anxiety, perceived social support, and COVID-19 related fear impacted speech-language, sensory-motor, and socio-emotional development in 12 months old Serbian infants during the COVID-19 pandemic.

**Methods:** This follow-up study included 142 pregnant women (Time 1) and their children at 12 months (Time 2). Antenatal maternal anxiety and children's development were examined. Maternal anxiety was assessed using the State-Trait Anxiety Inventory (STAI). Child speech-language, sensory-motor, and socio-emotional development were assessed using the developmental scale in the form of an online questionnaire that examined the early psychophysiological child development. Information on socioeconomic factors, child and maternal demographics, clinical factors, and perceived fear of COVID-19 viral infection were collected. Multivariable General Linear Model analysis was conducted, adjusted for demographic, clinical, and coronavirus prenatal experiences, maternal prenatal anxiety levels, perceived social support, speech-language, motor skills, and cognitive and socio-emotional development at the infants' age of 12 months.

**Results:** The study revealed the influence of the COVID-19 pandemic on maternal trait anxiety. The association between selected independent factors and infants' development was found in a demographically unified sample except for employment and the number of children. There was a correlation between all observed developmental functions. Univariate General Linear model statistical analysis indicated that linear models with selected independent factors and covariates could account for 30.9% (Cognition) up to 40.6% (Speech-language) of variability in developmental functions. It turned out that two-way and three-way interactions had a dominant role on models, and STAI-T Level and COVID-19 related fear were present in all interaction terms.

**Conclusion:** Our findings reveal important determinants of child developmental outcomes and underline the impact of maternal anxiety on early child development. These findings lay the groundwork for the following interdisciplinary research on pregnancy and child development to facilitate and achieve positive developmental outcomes and maternal mental health.

## Introduction

The conditions under which intrauterine development happens and the influences during childbirth and the postpartum period form the child's essential psychophysiological capacity. There is accumulating evidence about the importance of the first 1,000 days of life to a child's overall development (Black et al., 2017). During prenatal and early child development brain adapts in response to a wide range of early experiences, which supports the rapid acquisition of language, cognitive skills, and socioemotional competencies (Luby, 2015; Britto et al., 2017).

The prenatal period is the sensitive period for child development, in which negative associations between prenatal exposure to maternal anxiety and outcomes are observed (Comaskey et al., 2017). In this respect, perinatal maternal health may play an important role and influence the child's early development. A woman's mental health during pregnancy and the first year after birth refers to perinatal mental health. It includes mental health difficulties that occur before or during pregnancy and mental health problems that appear for the first time and can significantly increase during the perinatal period (Rees et al., 2019). Findings in the literature point to maternal mental well-being as crucial for optimal infant health (Ryan et al., 2017).

On the other hand, maternal mental health problems are considered a significant public health issue. Depression, anxiety, and high levels of perceived stress are the most common mental health problems during pregnancy (Hamid et al., 2008; Martini et al., 2015; Ryan et al., 2017; Rees et al., 2019).

Perinatal anxiety refers to anxiety experienced during the pregnancy and/or the first 12 months after birth. It may be significantly associated with postnatal anxiety (Grant et al., 2008). According to previous epidemiological studies, the prevalence of women who experienced high anxiety during pregnancy ranges from 6.8 to 59.5% (Leach et al., 2017). Research on the dynamics of the anxiety manifestation during pregnancy is inconsistent. While some authors reported a significant increase in anxiety during the last trimester of pregnancy (Gunning et al., 2010), others pointed to stable pregnancy-specific anxiety across all three trimesters of pregnancy (Rothenberger et al., 2011). In line with this, it is crucial to notice the difference between general anxiety and pregnancy-specific anxiety. These two entities are considered strictly interrelated, although the mechanisms of interrelation are not fully documented (Huizink et al., 2014). While pregnancy anxiety refers to an emotional state that is mostly situationally or contextually conditioned, general anxiety—trait anxiety, in particular, can be maintained and last in the period after pregnancy. In that way, general anxiety may continue to affect a mother's mental health and influence mother-child interaction (Huizink et al., 2014).

The impact of pregnant woman's anxiety on early child development has been a focus of recent studies. More specifically, interest in studying the link between prenatal anxiety and early childhood development has increased in the past 20 years. Van den Bergh et al. (2005) supported a fetal programming hypothesis and pointed out that the development of the hypothalamo–pituitary–adrenal (HPA) axis, limbic system, and the prefrontal cortex may be affected by maternal prenatal stress and anxiety. It results in cortisol passing through the placenta, affecting the fetus and disturbing ongoing developmental processes. Studies examining the impact of maternal postnatal anxiety on child development have indicated possible findings within three domains: somatic, developmental, and psychological outcomes (Glasheen et al., 2010). Similarly, studies examining the same outcomes about prenatal maternal anxiety suggest that maternal anxiety during pregnancy may also have long-lasting consequences on child development and behaviour (Dunkel Schetter and Tanner, 2012; Huizink et al., 2014). Research evidence indicates that high levels of maternal anxiety symptoms are associated with a wide range of adverse cognitive, behavioural, and neurophysiological offspring outcomes (O'Connor et al., 2014), as well as with temperamental and developmental problems (Hernández-Martínez et al., 2008). Infants of mothers with high trait anxiety have a predisposition to suboptimal nervous system development and may have an increased vulnerability for developing motor problems (Kikkert et al., 2010). On the other hand, research data on the direct impact of perinatal maternal anxiety on children's emotional problems lacks cohesion and indicate that maternal prenatal anxiety has a slight adverse effect on child emotional outcomes (Rees et al., 2019).

In addition, maternal anxiety in pregnancy is also associated with perceived social support (Sharif et al., 2021). Similarly to maternal anxiety, a pregnant woman's lack of perceived support may negatively affect her mental well-being, fetus, and close family members (Aktan, 2012). Social support plays a significant role in stressful life events and includes providing emotional, informational, and practical physical support (financial and material) during the time of need and within a person's social network (Dambi et al., 2018; Sharif et al., 2021). Research on social support during pregnancy has shown that it may be a strong predictor of a healthy pregnancy (Naveed et al., 2018). Several studies on the population of pregnant women found that social support plays a significant role in maternal well-being, while perceived lack of social support leads to mental health problems (Aktan, 2012; Denis et al., 2015; Sharif et al., 2021).

The mentioned aspects that may affect infant and child development need to be additionally considered to the coronavirus disease 2019 (COVID-19), which has grown into a global pandemic declared by the World Health Organization (WHO) on March 11, 2020 (Cucinotta and Vanelli, 2020). COVID-19 was reported for the first time in Wuhan, China, in December 2019, with increasing global transmission (Api et al., 2020). Data on the COVID-19 in the Republic of Serbia show that the first case of COVID-19 was reported on March 6, 2020, and soon after it, precisely on March 15, 2020, a state of emergency was declared in the whole country (Stašević-Karličić et al., 2020). Psychological impacts of COVID-19 on pregnancy have already been explored in recent studies (Quinlivan and Lambregtse-van den Berg, 2020; Usher et al., 2020; Yan et al., 2020; Motrico et al., 2021). An accompanying phenomenon of the COVID-19 pandemic is the fear of COVID-19 viral infection, which can intensify the level of anxiety (Andrade et al., 2020). Although the symptoms of anxiety manifested by individuals during the epidemic/pandemic may be similar to those expressed in other anxiety situations, it is noticed that there are specific forms of anxiety-related distress responses during viral outbreaks and the COVID-19 pandemic as well (Asmundson and Taylor, 2020). Some major factors which contribute to specificity of pandemic anxiety are the fear of becoming infected and dying, socially disruptive behaviors, and adaptive behaviors (Asmundson and Taylor, 2020; Bernardo et al., 2020; Wang et al., 2020). In other words, the increased risk of death (Aldridge et al., 2020), additionally unemployment and economic losses (Coibion et al., 2020), and numerous restrictions introduced by the countries' governments around the world, lead to negative health consequences (Meyerowitz-Katz et al., 2021), that include pandemic anxiety with its specifics as well (Bernardo et al., 2020). Accordingly, a fair number of studies have been published indicating an increase in anxiety symptoms in pregnant women during the COVID-19 pandemic (Ayaz et al., 2020; Corbett et al., 2020; Durankuş and Aksu, 2020; Kotabagi et al., 2020; Lebel et al., 2020; Moyer et al., 2020; Saccone et al., 2020; Wu et al., 2020; Yue et al., 2021). So far, there are studies, systematic reviews, and meta-analyses in which the prevalence of pregnant women with moderate to severe anxiety during the COVID-19 pandemic is given more precisely (Corbett et al., 2020; Hessami et al., 2020; Lebel et al., 2020; Mappa et al., 2020; Saccone et al., 2020; Fan et al., 2021; Sun et al., 2021; Tomfohr-Madsen et al., 2021). Accordingly, it ranges from: 23.4% found in Brazil (Nomura et al., 2021); the 29% was determined during the initial stage of the COVID-19 pandemic in China (Wang et al., 2020), while 25% was determined by meta-analysis (Ren et al., 2020); the 38% was found in Poland (Nowacka et al., 2021); the elevated level of the trait (38.2%) and state anxiety (77%) was found in Italy (Mappa et al., 2020). Higher prevalence in the range from 53 to 72% was found in other countries (Corbett et al., 2020; Dagklis et al., 2020; Davenport et al., 2020; Hessami et al., 2020; Lebel et al., 2020; Saccone et al., 2020; Sut and Kucukkaya, 2020). In contrast to these studies, a lower prevalence of anxiety among pregnant women (8.4% of whom had moderate anxiety and 5.2% of whom had severe anxiety) was determined in Belgium (Ceulemans et al., 2020), while COVID-19 did not increase anxiety levels in Dutch pregnant women (Zilver et al., 2021).

Generally, the analysis of the most recent systematic reviews and meta-analysis have shown that the prevalence of anxiety in pregnancy during COVID-19 ranges from 30.5 to 42%, the prevalence of depression ranges from 25 to 30%, and prevalence of both anxiety and depression was 18% (Fan et al., 2021; Sun et al., 2021; Tomfohr-Madsen et al., 2021).

Pregnant women may be affected by various aspects of COVID-19 pandemic that can cause negative implications on both maternal well-being and child development, which imposes the need for longitudinal studies of the COVID-19 birth cohort (Quinlivan and Lambregtse-van den Berg, 2020).

Given the clear need for longitudinal studies on the impact of maternal prenatal anxiety on early child development generally, and especially during the COVID-19 pandemic, the present prospective cohort study aimed to examine whether maternal trait anxiety, COVID-19 related fear and perceived social support were associated with speech-language, sensory-motor and socio-emotional development in 12 months old Serbian infants during COVID-19 pandemic. Demographic characteristics of mothers were also controlled in the analyses. Generally, it was hypothesized that maternal trait anxiety and COVID-19 related fear are prospectively and directly associated with infant development and may affect an infant speech-language, sensory-motor and socio-emotional development.

## Materials and Methods

### Study Design, Setting, and Participants

The present ongoing prospective cohort study is a part of a more extensive experimental study that examines maternal anxiety during pregnancy and its associated factors in the context of the COVID-19 pandemic in Serbia. This investigation examines the impact of maternal anxiety during pregnancy on early child development. Between April 2020 and December 2020, 900 pregnant women were included in the cohort. Expectant mothers were recruited consecutively, in the order in which they came for a regular examination during pregnancy on the Clinic for gynaecology and obstetrics “Narodni Front” in Belgrade. Women in the third trimester of pregnancy were asked to voluntarily fill out an anonymous self-administered questionnaire in a pleasant atmosphere in the waiting room during a time-optimal for them. The questionnaire contained socio-demographics, pregnancy-related background, maternal mental health, perceived social support, perceived COVID-19 related fear, and personal contact information related to an e-mail address and/or mobile phone. It is emphasized that personal contact information should be written if the pregnant woman agrees to provide data on her child development later in the longitudinal research. Of the total sample, 209 women did not complete the questionnaire, 187 women partially completed, while 145 women did not meet defined inclusion and exclusion criteria. The inclusion criteria for the study were: normal singleton pregnancy without complications of any kind; singleton pregnancies with the presence of hypertension, diabetes, or preterm delivery symptoms; spontaneous conception, delivering a phenotypically normal live birth, no pre-and perinatal risk factors. The exclusion criteria for the study were: failure to meet the inclusion criteria, infertility treatment; hospitalization; history of pre-eclampsia, eclampsia, autoimmune diseases, cancer, or any general chronic illnesses except hypertension or diabetes; psychiatric illnesses verified and/or treated before pregnancy; use of tranquilizers or sedatives, tobacco, alcoholic beverages, or any other type of psychoactive substances; non-acceptance of participation in the study. The final sample comprised 359 pregnant women in whom it was possible to examine the presence of anxiety during the COVID-19 pandemic in Serbia. All participants signed their written informed consent prior to the study, and confidentiality of the responses was assured.

**Figure 1:**
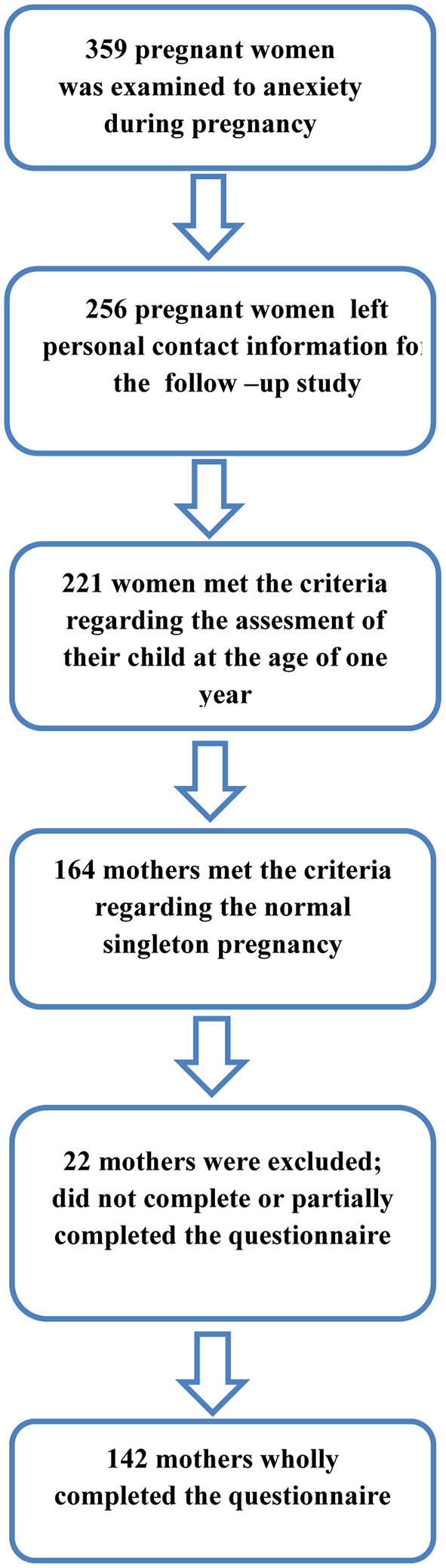
Definition of participation process by flow-chart.

The present study included a follow-up assessment of children aged 11.5 to 12.5 months whose mothers participated in a baseline assessment of maternal anxiety during the third trimester of pregnancy. Between May 2021 and September 2021, all mothers whose children aged 11.5 to 12.5 months were invited to participate in the study. Out of the sample of 359 pregnant women, 256 left personal contact information, while 221 of them met the criteria regarding the assessment of their children at the age of 1 year. In order to observe the possible influence of anxiety on early child development, we excluded mothers who had pregnancy complications. Thereby, the sample was reduced to 164 mothers who had normal singleton pregnancies without complications of any kind. Mothers were invited to complete an online questionnaire on their child's development by phone or e-mail. After collecting and analyzing the obtained data, the final analysis showed that 19 mothers did not complete, while three mothers partially completed the questionnaire related to child development. The final sample included *n* = of 142 mothers who completed the questionnaire related to child development (Figure 1). By completing the questionnaire, the mothers gave their consent to participate in the study. The sample was uniform with regard to all demographic factors except employment and the number of children.

The complete study protocol had been approved by the Ethics Committee of the Clinic for Gynecology and Obstetrics “Narodni Front” (Date: 26 March, 2020, No 27/20), in Belgrade, and by the Ethics Committee of the Institute for experimental phonetics and speech pathology (Date: 2 April, 2020, No 45/20), in Belgrade, which operates under the Ethical principles in medical research involving human subjects, established by the Declaration of Helsinki 2013.

### Measures

A self-administered anonymous questionnaire included questions related to socio-demographics, pregnancy-related background, maternal anxiety, perceived social support and perceived COVID-19 related fear.

*Maternal trait anxiety* was measured with the Spielberger State-Trait Anxiety Inventory (STAI), which is a frequently used measure for self-reported anxiety (Spielberger et al., 1983). Spielberger questionnaire form Y was used in our study (Spielberger et al., 2000). The STAI consists of two scales: state anxiety scale (STAI-S) and trait anxiety scale (STAI-T), each containing twenty items. STAI-S measures anxiety as a current state, while STAI-T measures anxiety as a personality trait, and it is considered to be more stable and more long-lasting (Easter et al., 2015). Since STAI-S is relatively transient and variable over time (Floris et al., 2017; Papadopoulou et al., 2017), it was not possible to conduct measurements by applying it in short time intervals during the COVID-19 pandemic, which would enable obtaining a reliable mean value of the current state of anxiety during the first year of a child's life. Concerning that, we evaluated only the STAI –T, as it measures relatively stable individual differences in propensity for anxiety (Julian, 2011). Participants select responses on a four-point Likert scale, ranging from “almost never,” “sometimes,” “often,” and “almost always.” The total score ranges between 20 and 80, with higher scores indicating greater anxiety levels. Some authors use cut-off points to define two-level state and trait anxiety (low and medium/high) (Özpelit et al., 2015; Mappa et al., 2020, 2021). We used three-level cut-off points for reasons described in the literature (Tomašević-Todorovic et al., 2012; Candelori et al., 2015). Accordingly, STAI-T scores were classified as “no or low anxiety” (20–30), “moderate anxiety” (31–44), and “high anxiety” (45–80).

*Maternal perecption of social support* was assessed with The Multidimensional Scale of Perceived Social Support (MSPSS) (Zimet et al., 1988), the Serbian version of the scale (Pejičić et al., 2018). MSPSS consists of twelve items divided into three subscales that measure the perception of social support from three sources: family, friends and significant others. Responses are rating on a seven-point Likert scale, ranging from 1 (“very strongly disagree”) to 7 (“very strongly agree”). The maximum score is 84 and indicates the highest degree of perceived social support. In the study, the total MSPSS score was used in the calculation and interpretation of the results.

*COVID-19-related fear* was assessed using a single item question “Do you feel a fear of COVID-19 viral infection?” Participants select one of three responses: 1–“I do not feel a fear of COVID-19 viral infection”; 2–“Sometimes, not all the time I feel a fear of COVID-19 viral infection”; and 3-”I do feel a fear of COVID-19 viral infection.”

*Language, sensory-motor and socio-emotional development* in 12 months old infants were assessed by The Scale for Evaluation of Psychophysiological Abilities of Children (Subota, 2003; Rakonjac et al., 2016; Vujović et al., 2019; Bogavac et al., 2021). The Scale for Evaluation of Psychophysiological Abilities of Children (SEPAC) is created according to developmental norms of the child from birth to 7 years. It comprises subtests specific for different months up to the first year of life and different years of age to the seventh year of life. Each subtest consists of three subscales: Speech-language scale, Sensory-motor scale and Socio-emotional scale. The speech-language scale consists of questions through which receptive speech, expressive speech and non-verbal communication are assessed. The sensory-motor scale consists of questions through which motor skills and cognition are assessed. Finally, the socio-emotional scale consists of questions through which a child's experience and self-regulation (child's social behaviour, emotional behaviour, regulation of attention, and thoughts) are assessed. The child's achievements within each scale are assessed with three possible answers: answer “+” indicates that your baby is performing the specified activity; answer “+/–” indicates that your baby sometimes or insufficiently performs an assessed activity, and answer “–” indicates that your baby is not yet performing an assessed activity. For the scoring of the test, answers marked with “+”are scored with 2 points, answers “+/–” with 1 point, and answers marked with “–” are scored with 0 points. In our study, we used a subtest that assesses the psychophysiological abilities up to 12 months of age. It consists of 43 simple, straightforward questions related to the assessment of the following abilities and skills: receptive and expressive speech (13), sensory-motor skills (9), cognition (12), and socio-emotional skills (9). The maximal number of points on subtests is 26, 18, 24, and 18, respectively.

### Statistical Analyses

Only women who completed the two questionnaires were included in the analyses. Descriptive statistics were used to determine central tendencies and distributions of variables. To determine the existence of relationships between variables, we conducted a bivariate correlation analysis. To investigate the effects of individual factors on the variables of interest and interactions between factors, we conducted Univariate General Linear Model Analysis. To perform hypothesis testing, a priori contrasts were applied and, depending on results *post-hoc* test. Statistical Package for the Social Sciences version 22.0 was used.

Before any statistical test, appropriate assumptions were checked. For STAI-T and MSPSS, we defined the new variables STAI-T level and MSPSS level, dividing the main variables into three groups. For STAI-T level, range limits are: if STAI-T is ≤30 Level is Low (1), if STAI-T is between 31 and 45 Level is Intermediate (2) and for values >45 Level is High (3). For MSPSS level range limits are: Low (1) <35; Medium (2) between 36 and 60; High (3) >61.

## Results

### Sample Characteristics

The final sample consisted of 142 mothers with a mean age of 29.56 years (SD = 4.88). The majority of participants (*n* = 88, 61.97%) had Bachelor's degree or higher, while 63.38% (*n* = 90) of them were employed. There were 50.70% (*n* = 72) mothers having one child and 49.30% (*n* = 70) having two or more children. Almost half of the participants (*n* = 64, 45.07%) reported having a COVID-19-related fear, 39.44% (*n* = 56) reported having no COVID-19 related fear, while 15.49% (*n* = 22) reported that they sometimes have a COVID-19 fear. Half of the participants (50.70%, *n* = 72) had an intermediate level of anxiety, while 49.30% (*n* = 70) had a high level of anxiety measured on STAI-T. The vast majority of participants (81.69%, *n* = 116) had an intermediate level of anxiety, while 18.31% (*n* = 26) had a high level of anxiety measured on STAI-S. All participants reported a high level of perceived social support. Baseline sample characteristics are shown in Table 1.

**Table 1:** Sample Description (*N* = 142).

**Sample characteristics**	**Mothers (*N* = 142) Mean (SD) or (%)**	
Maternal age (years**)**	29.56	(4.88)
**Years of school education**		
<12 years	88	(61.97)
12 or more years	54	(38.03)
**Employment status**		
Employed	90	(63.38)
unemployed	52	(36.62)
**Number of children**		
One child	72	(50.70)
Two or more children	70	(49.30)
**COVID-19-related fear**		
Having a COVID-19-related fear	64	(45.07)
Having no COVID-19 related fear	56	(39.44)
Sometimes have a COVID-19 fear	22	(15.49)
**STAI-T**		
Low anxiety level	–	–
Intermediate anxiety level	72	(50.70)
High anxiety level	70	(49.30)
**STAI-S**		
Low anxiety level	–	–
Intermediate anxiety level	116	(81.69)
High anxiety level	26	(18.31)
**MSPSS**		
Low perceived support	–	–
Medium perceived support	–	–
High perceived support	142	(100)

The average STAI-T score was 44.59 ± 4.61. As previously mentioned (Table 1), no participants had scores corresponding to low anxiety levels. When observing the findings of social support, it is noticed that the average MSPSS score was 76.70 ± 7.14. Results related to infant's achievement included the estimation of speech-language development, sensory-motor development in which the development of motor skills and cognition was observed as two separate variables, and socio-emotional development. The average speech-language score was 20.27, which is 77.9% of maximal achievement score; the average motor skills score was 13.33, which is 74.06% of maximal achievement score; the average cognition score was 18.99, which is 79.12% of maximal achievement score, and the average socio-emotional score was 14.22 which is 79% of maximal achievement score (Table 2).

**Table 2:** Descriptive statistics on maternal anxiety, perceived social support, and infants' achievement.

**Variable**	**Min**.	**Max**.	**Mean**	**SE**	**SD**
Maternal age	19.00	39.00	29.56	0.409	4.88
STAI-T	31.00	55.00	44.59	0.387	4.614
MSPSS	62.00	84.00	76.70	0.599	7.137
Speech-language development	12.00	26.00	20.27	0.302	3.602
Motor skills	4.00	18.00	13.33	0.296	3.524
Cognition	11.00	24.00	18.99	0.304	3.619
Socio-emotional development	7.00	18.00	14.22	0.246	2.930

### One-Way Anova

Comparing mean values for STAI-T and MSPSS related to fear of getting COVID-19 (Table 3) shows that mean values are not equal.

**Table 3:** Descriptive statistics for STAI-T and MSPSS related to COVID-19 related fear.

	**COVID-19 related fear**	***N***	**Mean**	**SD**	**SE**
STAI-T	No	64	43.53	4.75	0.594
	Sometimes	56	45.46	3.95	0.528
	Yes	22	45.45	5.30	1.129
MSPSS	No	64	73.84	7.77	0.972
	Sometimes	56	79.11	5.13	0.6868
	Yes	22	78.91	6.80	1.449

One-Way ANOVA statistical test was used to check the impact of COVID-19 related fear on STAI-T and MSPSS. For STAI-T, homogeneity of variance is not violated, and *post-hoc* test with Tukey-Kramer correction for multiple comparisons (uneven sample size) was used. The MSPSS Games-Howell method was used because of its robustness when homogeneity of variance is violated, and the sample size is unequal.

There was statistically significant difference for STAI-T *F*_(2,139)_ = 3.171, *p* = 0.045. *Post-hoc* test revealed that there was no statistically significant difference between groups.

It was found that there is a statistically significant difference for MSPSS between the observed groups *F*_(2,139)_ = 10,646, *p* < 0.001. *Post-hoc* test showed statistically significant difference between groups 1 and 2 (*p* = 0.016), 1 and 3 (*p* < 0.001) and that there was no statistically significant difference between groups 2 and 3 (*p* = 0.992).

Mothers who reported no COVID-19 related fear have lower perceived social support (mean value 73.8) than mothers in the other two groups (Table 3).

### Correlations

Bivariate correlation analysis revealed a statistically significant correlation between Speech-language and Motor skills, Cognition and Socio-emotional status (Table 4). The highest correlation is between Speech-language and Socio-emotional status [r_(140)_ = 0.744, *p* < 0.001]. Also, there is a low but statistically significant correlation between the child's socio-emotional status and maternal trait anxiety r_(140)_ = 0.184, *p* = 0.028.

**Table 4:** Pearson correlation between variables of interest.

		**Speech-language**	**Motor skills**	**Cognition**	**Socio-Emotional status**
Speech-Language	Pearson corr.	1	0.273^**^	0.544^**^	0.744^**^
	Sig. (2-tailed)		0.001	0.000	0.000
Motor skills	Pearson corr.	0.273^**^	1	0.379^**^	0.276^**^
	Sig. (2-tailed)	0.001		0.000	0.001
Cognition	Pearson corr.	0.544^**^	0.379^**^	1	0.698^**^
	Sig. (2-tailed)	0.000	0.000		0.000
Socio-Emotional status	Pearson corr.	0.744^**^	0.276^**^	0.698^**^	1
	Sig. (2-tailed)	0.000	0.001	0.000	
Maternal age	Pearson correlation	−0.007	0.046	−0.035	−0.079
	Sig. (2–tailed)	0.938	0.590	0.682	0.353
STAI-T	Pearson correlation	0.163	−0.108	0.159	0.184^*^
	Sig. (2-tailed)	0.052	0.203	0.059	0.028
MSPSS	Pearson correlation	0.122	0.150	0.091	0.019
	Sig. (2-tailed)	0.149	0.075	0.283	0.825

*Significant correlations are marked in grey*.

* *significance at the level p < .05*.

** *significance at the level p ≤ . 001*.

The association between factors and developmental abilities (Table 5) ranges between weak and medium. If the mean value of eta for all developmental functions is observed to assess the association of individual factors and the child's overall development, then that association is at the level of medium association for STAI-T and weak for the other three factors.

**Table 5:** Level of association between development abilities and factors.

**Developmental function**	**Level of association** **η**			
	**STAI-T level**	**COVID fear**	**Number of children**	**Employment**
Communication	0.405	0.475	0.371	0.432
Motor skills	0.449	0.277	0.331	0.449
Cognition	0.515	0.313	0.353	0.266
Socio emotional	0.254	0.378	0.415	0.363
Mean value	0.40575	0.36075	0.3675	0.3775

### Multifactor Analysis

It was not possible to model the relationship between children developmental abilities (Speech-language, Motor skills, Cognition and Socio-emotional status) and STAI-T, MSPSS, COVID-19 related fear, employment, maternal age and number of children that mother has, with multivariate GLM because Box's Test revealed that Equality of Covariance Matrices across groups is violated. Box's M = 359.32, *F*_(100,5,441.018)_ = 2.833, *p* < 0.001. Levene's Test of Equality of Error Variances revealed that the assumption of equality is not violated, so we conducted separate univariate GLM tests where dependent variables were Speech-language, Motor skills, Cognition and Socio-emotional status with factors: employment, number of children, COVID-19 related fear, and STAI-T level. Mother age and MSPSS were included in the model as covariates.

To determine if there was statistically significant difference between mean values of groups within interaction terms, new grouping variables were composed for each interaction term (Appendix A). One–way ANOVA for dependent variables was conducted. Only interaction terms of dependent variables with Observed Power >0.8 were considered.

#### Results of Speech-Language Achievement

Univariate GLM analysis for dependent variable SPEECH-LANGUAGE achievement revealed that full linear model (Appendix B) could explain 40.6% of variability *F*_(21,120)_ = 3.902, *p* < 0.001, ηp^2^ = 0.406 and Observed Power = 1. Maternal age and Employment as a main effects have statistically significant impact on the model. Impact of the Employment can't be analyzed separately because of its interaction with COVID-19 related fear and STAI-T level. The biggest contribution to model has interaction term Number of children ^*^ COVID-19 related fear ^*^ STAI-T level (Partial eta squared = 0.182).

One-way ANOVA revealed that there was no statistically significant difference between groups of interaction terms Employment ^*^ COVID-19 related fear [*F*_(5,136)_ = 2.034, *p* = 0.078] and Employment ^*^ STAI-T level [*F*_(3,138)_ = 2.199, *p* = 0.091] but for interaction term Number of children ^*^ COVID-19 related fear ^*^ STAI-T level, there is statistically significant difference between groups *F*_(11,130)_ = 2.839, *p* = 0.002. *Post-hoc* test with Tukey-Kramer correction was used. It turns out that group of children whose mothers have one child, sometimes fearing getting COVID-19 infection and intermediate level of STAI-T (group 2) have the highest level of speech-language achievement (mean value = 22.67). There is statistically significant difference between this group and group 4 (p2–4 = 0.006). On the other hand, children groups 3, 4, and 12 have minimal speech-language achievements (17.50, 17.56, 17.50, respectively), but there were no statistically significant differences between group mean values except mentioned between groups 2 and 4. Explanation of membership to the variable group is given in Table c in Appendix A.

**Figure 2:**
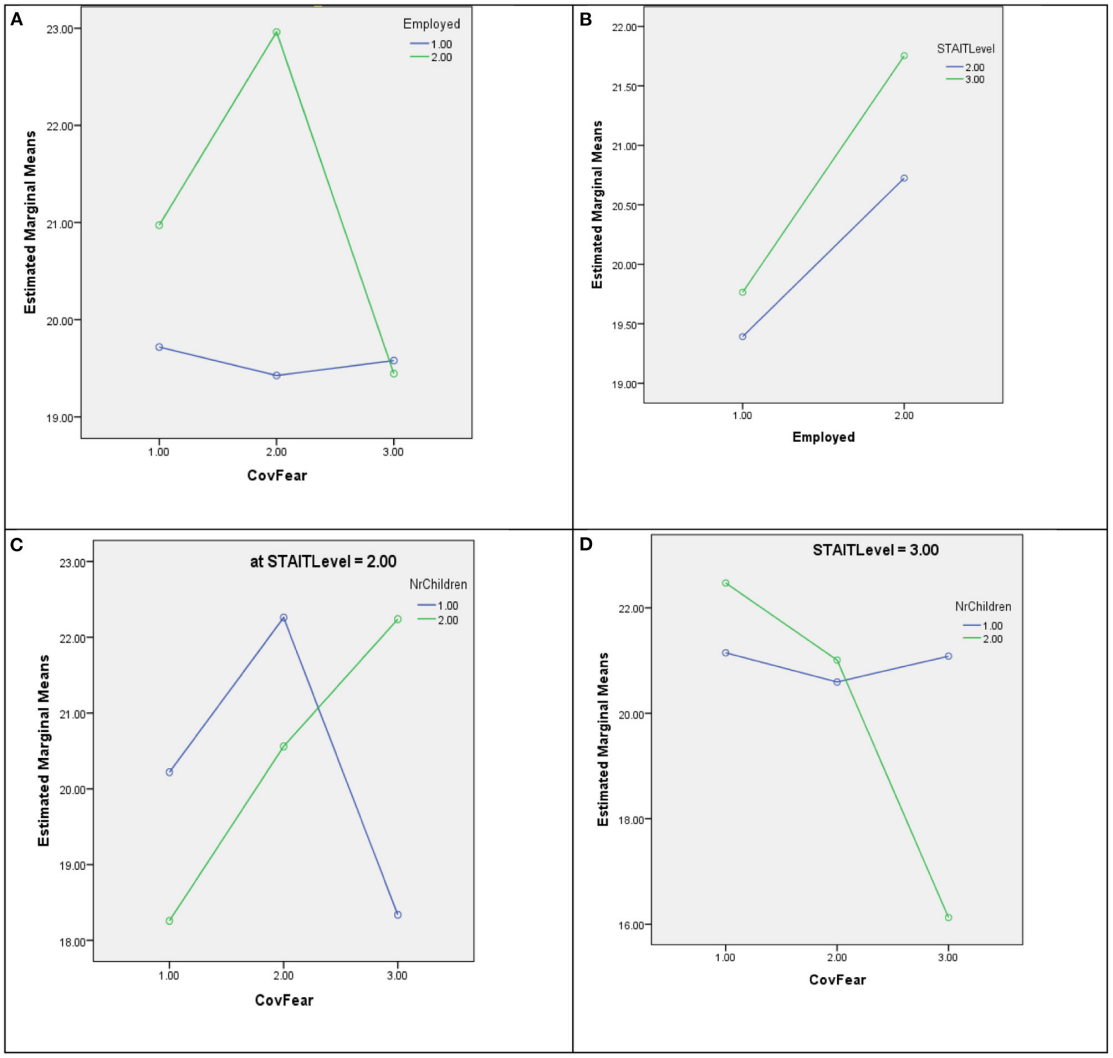
Plot of estimated marginal means of dependent variable speech-language for interactions **(A)** Employed * COVID-19 related fear, **(B)** Employed * STAI-T level, **(C)** Number of children * COVID-19 related fear * STAI-T level where STAI-T level = 2, and **(D)** Number of children * COVID-19 related fear * STAI-T level where STAI-T level = 3. Covariates appearing in the model are evaluated at the following values: Maternal age = 29.5634, MSPSS = 76.7042.

In Figure 2, the plot of estimated marginal means of dependent variable Speech-language for statistically significant interactions is presented as an example of interaction terms in all four univariate GLM models.

#### Results of Motor Skills Achievement

Univariate GLM analysis for dependent variable MOTOR skills achievement revealed that the full linear model (Appendix B) could explain 34.7% of variability *F*_(21,120)_ = 3.031, *p* < 0.001, ηp^2^ = 0.347 and Observed Power = 0.999. One-way ANOVA for interaction term Employed ^*^ COVID-19 related fear ^*^ STAI-T level revealed statistically significant difference between groups *F*_(11,130)_ = 2.206, *p* = 0.018. It turns out that children from group 6 had the highest (mean value = 16.00), and children group 11 had the lowest level (mean value 11.42) of Motor skills achievement. *Post-hoc* test with Tukey-Kramer correction revealed that there was no statistically significant difference between particular groups. Explanation of membership to the variable group is given in Table d in Appendix A.

Univariate GLM analysis for dependent variable COGNITION achievement revealed that full linear model (Appendix B) could explain 30.9% of variability *F*_(21,120)_ = 2.555, *p* = 0.001, ηp^2^ = 0.309 and Observed Power = 0.997.

One-way ANOVA revealed a statistically significant difference between groups of interaction term Employed ^*^ COVID-19 related fear [*F*_(5,136)_ = 2.825, *p* = 0.017]. *Post-hoc* test with Tukey-Kramer correction was used. It turns out that group 5 has the highest level of cognitive achievement (mean value = 21.5). There is statistically significant difference between this group and groups 3 and 4 (p5–3 = 0.016, p5–4 = 0.021). On the other hand, children group 3 has minimal Cognitive achievement (mean value = 17.44), but there was no statistically significant difference between group mean values except mentioned between groups 3 and 5. Explanation of membership to the variable group is given in Table a in Appendix A.

For interaction term Number of children ^*^ COVID-19 related fear ^*^ STAI-T level, we obtained statistically significant difference between groups *F*_(11,130)_ = 2.266, *p* = 0.015. It turns out that children group 10 had the highest (mean value = 20.625), and children group 12 had the lowest level (mean value 15.25) of Cognition achievement. *Post-hoc* test with Tukey-Kramer correction revealed that there is statistically significant difference between groups 10 and 4. Children group 4 had a mean value of cognition achievement of 16.190. This finding is a bit specific, but in that group were 16 samples while in group 12 were only 4. Explanation of membership to the variable group is given in Table c in Appendix A.

#### Results of Socio-Emotional Achievement

Univariate GLM analysis for dependent variable SOCIO-EMOTIONAL achievement revealed that the full linear model (Appendix B) could explain 39.1% of variability *F*_(21,120)_ = 3.673, *p* < 0.001, ηp^2^ = 0.391 and Observed Power = 0.999.

One-way ANOVA revealed a statistically significant difference between groups of interaction term Employed ^*^ STAI-T level [*F*_(3,138)_ = 3.309, *p* = 0.022]. *Post-hoc* test with Tukey-Kramer correction was used. It turns out that group 4 has the highest level of Socio-emotional achievement (mean value = 15.54). There is statistically significant difference between this group and group 1 (p4–1 = 0.011). On the other hand, children group 1 has minimal Socio-emotional achievement (mean value = 13.40), but there was no statistically significant difference between group mean values except mentioned between groups 1 and 4. Explanation of membership to the variable group is given in Table b in Appendix A.

For interaction term Number of children ^*^ COVID-19 related fear ^*^ STAI-T level, there was no statistically significant difference between groups *F*_(11,130)_ = 1.811, *p* = 0.058. It turns out that children group 2 had the highest (mean value = 16.0), and children group 3 had the lowest level (mean value 12.33) of Socio-emotional achievement. Explanation of membership to the variable group is given in Table c in Appendix A.

## Discussion

The present prospective cohort study examined whether maternal trait anxiety was associated with speech-language, sensory-motor and socio-emotional development in 12 months old Serbian infants during COVID-19 pandemic. Prenatal maternal anxiety may represent a relevant risk factor that interferes in various ways with child development. There is already quite a bit of evidence on the consequences that prenatal maternal anxiety may produce on the psychophysiological child development (Dunkel Schetter and Tanner, 2012; Huizink et al., 2014; O'Connor et al., 2014; Rees et al., 2019), while the interest in this topic has increased significantly in the past 20 years. The impact of maternal prenatal anxiety on child development needs to be additionally considered to the COVID-19 pandemic, which impacts on pregnancy and maternal mental health have also been studied in the past 2 years (Quinlivan and Lambregtse-van den Berg, 2020; Usher et al., 2020; Yan et al., 2020; Motrico et al., 2021). On the other hand, there are scarce data about longitudinal trajectories of maternal prenatal anxiety on early child development during the COVID-19 pandemic (Barišić, 2020; Quinlivan and Lambregtse-van den Berg, 2020; Araújo et al., 2021).

The present study sought to explore whether maternal prenatal anxiety impacts psychophysiological development in 12 months old Serbian infants during the COVID-19 pandemic. Specifically, the aim was to investigate the prospective impact of maternal prenatal anxiety on infant speech-language, sensory-motor and socio-emotional development at the age of 12 months, controlling maternal age, employment status, the number of children that mother has, COVID-19-related fear, STAI-T, and MSPPS.

Our main findings were as follows: Pregnant women from the sample, who were examined during the third trimester of pregnancy, had intermediate and high levels of trait anxiety, while none had low levels of anxiety. Also, all pregnant women reported a high level of perceived social support. Almost half of the pregnant women reported a COVID-19-related fear, while a group of pregnant women whose number is slightly less than half reported that they have no COVID-19 related fear. Only a small percentage of pregnant women reported that they sometimes had COVID-19-related fear. Moreover, pregnant women who reported having a COVID-19 related fear had higher trait anxiety levels. The average developmental achievements in infants aged 12 months were as follows: the highest level of achievement was present in the assessment of cognition, followed by average achievement in socio-emotional development, then in speech and language development, and finally in motor skills. The experimental factors have an impact on infants' development.

### Maternal Trait Anxiety During Pregnancy in the Context of COVID-19 Pandemic

The study found intermediate and high levels of maternal anxiety among 142 pregnant women from Serbia. The same findings were observed both on STAI-S and STAI-T scale. It was noticed that none of the study participants had a low level of maternal anxiety during pregnancy. In further analysis of the results, we observed the values of STAI-T, which measure relatively stable individual differences in propensity for anxiety (Julian, 2011), while the STAI-S values are relatively transient and variable over time (Floris et al., 2017; Papadopoulou et al., 2017). Similarly to findings from the literature relating to the COVID-19 pandemic and mental health consequences (Shigemura et al., 2020; Xiang et al., 2020), and especially the consequences on maternal mental health (Corbett et al., 2020; Mappa et al., 2020; Saccone et al., 2020; Yan et al., 2020), our findings also indicated that the COVID-19 pandemic has a profound impact on maternal prenatal anxiety, and maternal mental health in general. Various factors during the COVID-19 pandemic have been identified that lead to mental health consequences (Berthelot et al., 2020; Liu et al., 2021). However, the exact prevalence of anxiety among pregnant women during the COVID-19 pandemic is currently unknown, although recent research from different countries suggests elevated symptoms of anxiety in this population of women (Liu et al., 2021; Tomfohr-Madsen et al., 2021). Considering the above data on elevated anxiety symptoms in pregnancy during the COVID-19 pandemic, we could partly explain only the participation of pregnant women with intermediate and high levels of anxiety and the absence of pregnant women with low levels of anxiety in our study. On the other hand, all pregnant women were examined in the third trimester of pregnancy, which emerged as the most vulnerable to the manifestation of high anxiety levels compared to the previous two trimesters (Gunning et al., 2010).

### The Role of Perceived Social Support

The present study revealed that all pregnant women reported increased social support during the COVID-19 pandemic, which is in line with recent studies (Zhang and Ma, 2020; Hashim et al., 2021). This could be explained by the burdensome circumstances that resulted from the COVID-19 pandemic, since social support acts as a protective factor against the adverse mental health difficulties resulting from epidemics and natural disasters (King et al., 2012), and has been identified as an essential protection against stressful life events (Dambi et al., 2018).

Though certain studies indicate that there is a significant inverse relationship between social support and state and trait anxiety in pregnancy (Aktan, 2012), even during the COVID-19 pandemic (Lebel et al., 2020; Khoury et al., 2021), we did not notice such a relationship in our study, but the opposite one. This might be explained by the hypothesis that explains how the impact of social support on health increases during stressful circumstances (Flannery and Wieman, 1989). On the other hand, although there is large evidence that social support predicts depression and vice versa, there is little evidence to explain the directionality of perceived social support and anxiety (Dour et al., 2014). Especially, there are no precise indicators on the impact on individuals' social aspect and the mental health of pregnant women during the COVID-19 epidemic (Api et al., 2020). Findings from our study showing a high level of perceived social support in pregnant women, despite their high level of anxiety during the COVID-19 pandemic, could be further explained by the role that social support has as an essential coping resource, as well as a mechanism for the maintenance of psychological well-being under conditions of psychological burdens (Bruwer et al., 2008). Also, it is important to consider the fact that there is a strong family bonding in Serbian culture, especially during women's pregnancy when family and friends give strong support to women in every sense.

### The Role of COVID-19 Related Fear

Although resent research indicated that the COVID-19 pandemic might cause a significant long-lasting increase in fear (Skoda et al., 2020), in our study, less than half of pregnant women reported having a fear of getting a coronavirus infection (COVID-19-related fear). Our results should be interpreted in line with findings that point to decreasing of COVID-19 fear within 6 weeks from the pandemic outbreak (Hetkamp et al., 2020), but also with evidence indicating that individuals may respond differently to the emotional distress caused by traumatic events such as this pandemic (Killgore et al., 2021).

Pregnant women who reported that they have no COVID-19 related fear had lower STAI-T anxiety levels. One-way ANOVA showed a statistical significance of the difference in average STAI-T values to the presence of COVID-19 related fear. However, *post-hoc* analysis subsequently showed that there was no statistical significance between the groups. However, such findings indicating a connection between fear and trait anxiety are in line with the literature that confirmed this relationship (Paredes et al., 2021).

### Developmental Achievements of Infants Aged 12 Months

Child development is a complex maturational and interactive process that includes a gradual progression of perceptual, motor, cognitive, language, socio-emotional and self-regulatory abilities (Sameroff, 2009). There is increasing evidence of the importance of the first 1,000 days of life on later human development (Black et al., 2017; Agarwal et al., 2020). In that light, we also observed the first 12 months of the child's development, the age of the examined children in our study.

Harmonization of developmental abilities is a precondition for orderly child development (Sameroff, 2009). Observing the developmental abilities of infants aged 12 months in our study, we noticed that the highest level of correlation exists between socio-emotional development and speech-language development; then between socio-emotional development and cognition; then between cognition and motor skills, and finally between speech-language and motor skills. Some authors pointed to the connection between the mentioned abilities, which is not simple and direct, but rather complex and multiple (Piek et al., 2008; Wang et al., 2014; Bedford et al., 2016). The links between achievements in cognitive, social communication, and language development were noted, considering the assumed correlation and interdependence of the mentioned abilities (Iverson, 2010; Libertus and Violi, 2016). The highest association between socio-emotional development and speech-language development found in this study may be explained by the close connection between emotion and action, within which communication has a central aspect of action (Saarni et al., 2006). The quality of the maternal-infant relationship has a significant influence on infant development (Johnson, 2013). On the other hand, some authors pointed that a mother's emotional connection with her child has an essential role in predicting social–emotional outcomes and less cognitive, language, and motor development outcomes in infant development (Le Bas et al., 2021). We assumed that maternal-infant bonding, as one of the predictors of child development (Johnson, 2013), has a significant role even more during the COVID-19 pandemic, and thus reflects infants' socio-emotional development, and speech-language consequently.

The relationship between motor skills and language, cognitive, social, and perceptual development has been intensively studied in recent years (Leonard and Hill, 2014; Leonard et al., 2015; Libertus and Violi, 2016; Libertus and Hauf, 2017; Collett et al., 2019). Interactions between cognition, language, and speech motor skills at an early developmental stage are also shown (Nip et al., 2011). However, the weakest correlation between speech-language and motor skills found in this study may be interpreted with similar findings (Alcock and Krawczyk, 2010), although it is not yet fully understood how the development of motor skills affects the development of speech-language (Libertus and Violi, 2016).

### The Potential Impact of Maternal Trait Anxiety on the Early Infants' Development During COVID-19 Pandemic

Bearing in mind that in our study, all mothers during pregnancy had an intermediate or high level of trait anxiety, it was important to examine the impact of anxiety on the early infants' development. The impact of pregnant woman's anxiety on early child development has been well-documented (O'Connor et al., 2002, 2014; Dunkel Schetter and Tanner, 2012; Huzinik, 2014). Also, an essential issue under consideration is the impact of the COVID-19 pandemic upon pregnancy, childhood and adult outcomes (Quinlivan and Lambregtse-van den Berg, 2020). The results of our study pointed to a weak positive correlation between maternal trait anxiety and the child's socio-emotional status, which is in line with the literature (Rees et al., 2019).

In addition, literature data have shown that other factors may also affect the child's development, such as the maternal age, employment, and the number of children the mother has (Brooks-Gunn et al., 2002; Bernal, 2008; Chittleborough et al., 2011; Tearne, 2015; Duncan et al., 2018; Falster et al., 2018). With this in mind, we decided to examine the influence of these factors depending on the levels of trait anxiety, COVID-19 related fear, and perceived social support.

Table 5 shows the association between the independent factors STAI_T level, COVID-19 related fear, Number of children and Employment (nominal type) and the dependent variables Speech and language, Motor skills, Cognition and Socio-emotional achievement (scale type). It is evident that association depends on factor variable combination. For example, STAI-T level has the highest association with Cognition (η = 0.515) and the lowest with Socio-emotional achievement (η = 0.0.254), while Employment has the highest association with Motor skills (η = 0.449) and the lowest with Cognition (η = 0.0.266) It is important to notice that all independent factors are associated with child's development. Results indicate that part of the variability of developmental abilities can be explained by factors. It implies that the factors have an impact on child development.

Univariate analysis showed that between 30.9 and 40.6% variability of dependent variables Speech-language, Motor skills, Cognition and Socio-emotional achievement could be explained by factors Employed, Number of children, COVID-19 related fear and STAI-T level and covariates Maternal age and MSPSS. It can be concluded from Table 5 that interaction terms have a dominant impact on models. In all models statistically significant impact of COVID-19 related fear and STAI-T level interaction is present. However, this two-way interaction also occurs as an element of a statistically significant three-way interaction. In the case of all dependent variables, they occur in interaction with number of children. Only in Motor skills Observed Power is <0.8 (0.741). In the case of Motor skills, three-way interaction with Observed Power >0.8 (0.927) occurs between COVID-19 related fear, STAI-T level, and Employed. The interaction of factors and their influence can be easily observed from the example given in Figure 2.

If we look at the three-way interaction term Number of children ^*^ COVID-19 related fear ^*^ STAI-T level found in all four linear models, we notice that the influence of individual factors on achievements within the developmental functions is different. That is, for each of the four development functions, the maximum and minimum achievements are influenced by different combinations of interacting factors values. For example, while children of mothers who have one child are sometimes afraid of COVID-19 and have an intermediate level of STAI-T have the most developed speech and language, children of mothers who have two or more children have no fear of COVID-19 and have a high level of STAI-T show the best cognitive abilities. The obtained results indicate a complex interdependence of the observed factors and their influence on the development of the observed functions in the first 12 months of a child's life. We can say that the mother-child relationship in this period is subject to the influence of various factors, reflecting on the child's development and having complex implications on individual functions.

The conducted research did not answer the question of the individual influence of the analysed factors on the child's development, but it confirmed our hypothesis that the mother's anxiety affects the child's development. COVID-19 related fear has also been shown to have an effect. In that sense, considering that there are no consolidated studies on the possible impacts of COVID-19 on pregnant women's health and their children's development, especially over the long term, maternal anxiety during this period is a problem that needs to be more widely addressed.

## Conclusion

The study showes that the COVID-19 pandemic affects the level of trait anxiety manifestation in pregnant women and the level of perceived social support. Selected factors as follows: maternal age, the level of maternal anxiety, and the maternal COVID-19 related fear influence the infants' development. This study confirmes our hypothesis that maternal anxiety and fear of COVID-19 have an influence on infant's development. Due to the interaction between the factors, their individual influence could not be precisely determined. Further focused research should provide an answer to this question.

Generally, this study aims to improve the understanding of the impact of the COVID-19 pandemic on infants' development, which can help to guide appropriate strategies to prevent dysfunctions in the children's development on the one hand, and on the other to promote a stimulating environment for children.

### Strengths and Limitations

To the best of our knowledge, there are no studies that monitor the early infant's development under the influence of the mother's mental health during the COVID-19 pandemic. Since this study was conducted during a COVID-19 pandemic, we assume that it resulted without having a group of mothers with a low level of trait anxiety. In that sense, it was not possible to make a more precise conclusion about the influence of maternal anxiety on the infants' development during the COVID-19 pandemic. On the other hand, it is not possible to accurately state the impact of each observed factor on the early infants' development. It is also important to note that the comorbidity of depression/anxiety is very high, and its impact on child's development during the COVID-19 pandemic was not estimated in our study. Also, only the mothers were included in the study, which indicates that without including fathers/partners, the impact of child development could not be determined accurately. The findings from this study suggest that future research is warranted to investigate the longitudinal impacts of maternal and parental mental health on child development during the COVID-19 pandemic, taking into account additional measurement points and modifiable risk factors. The need for systematic monitoring of children at the earliest age was pointed out, and providing the necessary support to pregnant women and fathers/partners during the COVID-19 pandemic.

## Author's Note

LJ, MSo, OG, and MK are management committee members of COST Action CA18211: DEVoTION: Perinatal Mental Health and Birth-Related Trauma: Maximizing best practice and optimal outcomes. This paper contributes to the EU COST Action 18211: DEVoTION.

## Data Availability Statement

The datasets presented in this article are not readily available because of legal and ethical constraints. Public sharing of participant data was not included in the informed consent of the study. Requests to access the datasets should be directed to Miško Subotić, m.subotic@add-for-life.com.

## Ethics Statement

The studies involving human participants were reviewed and approved by Ethics Committee of the Clinic for Gynecology and Obstetrics Narodni Front (Date: 26 March, 2020, No 27/20), in Belgrade and by the Ethics Committee of the Institute for experimental phonetics and speech pathology (Date: 2 April, 2020, No 45/20), in Belgrade, Serbia. Written informed consent to participate in this study was provided by the participants' legal guardian/next of kin.

## Author Contributions

LJ and MSu: conceptualization and visualization. LJ and MSo: methodology. MSu: formal, statistical analysis, and supervision. LJ, IB, and AD: investigation. AD and IB: data curation. LJ: supervision of the (ongoing) data collection and writing—original draft preparation. MSo, OG, and MK: writing—review and editing. All authors contributed to the article and approved the submitted version.

## Conflict of Interest

The authors declare that the research was conducted in the absence of any commercial or financial relationships that could be construed as a potential conflict of interest. The reviewer ML declared a shared affiliation, with no collaboration, with one of the authors MK to the handling editor at the time of the review.

## Publisher's Note

All claims expressed in this article are solely those of the authors and do not necessarily represent those of their affiliated organizations, or those of the publisher, the editors and the reviewers. Any product that may be evaluated in this article, or claim that may be made by its manufacturer, is not guaranteed or endorsed by the publisher.

## References

[B1] Agarwal P. K. Xie H. Rema A. S. S. Rajadurai V. S. Lim S. B. Meaney M.. (2020). Evaluation of the ages and stages questionnaire (ASQ 3) as a developmental screener at 9, 18, and 24 months. Early Hum. Dev. 147:105081. 10.1016/j.earlhumdev.2020.105081 32502946

[B2] Aktan N. M. (2012). Social support and anxiety in pregnant and postpartum women: a secondary analysis. Clin. Nurs. Res. 21, 183–194. 10.1177/1054773811426350 22019712

[B3] Alcock K. J. Krawczyk K. (2010). Individual differences in language development: relationship with motor skill at 21 months. Dev. Sci. 13, 677–691. 10.1111/j.1467-7687.2009.00924.x 20712734

[B4] Aldridge R. W. Lewer D. Katikireddi S. V.. (2020). Black, Asian and Minority Ethnic groups in England are at increased risk of death from COVID-19: indirect standardisation of NHS mortality data [version 2; peer review: 3 approved]. Wellcome Open Res. 5:88. 10.12688/wellcomeopenres.15922.2 32613083 PMC7317462

[B5] Andrade E. F. Pereira L. J. Oliveira A. P. L. D. Orlando D. R. Alves D. A. G. Guilarducci J. D. S.. (2020). Perceived fear of COVID-19 infection according to sex, age and occupational risk using the Brazilian version of the fear of COVID-19 scale. Death Stud. 1–10. 10.1080/07481187.2020.1809786 32845795

[B6] Api O. Sen C. Debska M. Saccone G. D'Antonio F. Volpe N.. (2020). Clinical management of coronavirus disease 2019 (COVID-19) in pregnancy: recommendations of WAPM-World Association of Perinatal Medicine. J. Perinat. Med. 48, 857–866. 10.1515/jpm-2020-0265 32692708

[B7] Araújo L. A. D. Veloso C. F. Souza M. D. C. Azevedo J. M. C. D. Tarro G. (2021). The potential impact of the COVID-19 pandemic on child growth and development: a systematic review. J. Pediatr. 97, 369–377. 10.1016/j.jped.2020.08.008 32980318 PMC7510529

[B8] Asmundson G. J. G. Taylor S. (2020). How health anxiety influences responses to viral outbreaks like COVID-19: What all decision-makers, health authorities, and health care professionals need to know. J. Anxiety Disord. 71:102211. 10.1016/j.janxdis.2020.102211 32179380 PMC7271220

[B9] Ayaz R. Hocaoglu M. Günay T. Devrim Yardimci O. Turgut A. Karateke A. (2020). Anxiety and depression symptoms in the same pregnant women before and during the COVID-19 pandemic. J. Perinat. Med. 48, 965–970. 10.1515/jpm-2020-0380 32887191

[B10] Barišić A. (2020). Conceived in the covid-19 crisis: impact of maternal stress and anxiety on fetal neurobehavioral development. J. Psychosom. Obstet. Gynaecol. 41:246. 10.1080/0167482X.2020.1755838 32326793

[B11] Bedford R. Pickles A. Lord C. (2016). Early gross motor skills predict the subsequent development of language in children with autism spectrum disorder. Autism Res. 9, 993–1001. 10.1002/aur.1587 26692550 PMC5031219

[B12] Bernal R. (2008). The effect of maternal employment and child care on children's cognitive development. Int. Econ. Rev. 49, 1173–1209. 10.1111/j.1468-2354.2008.00510.x

[B13] Bernardo A. B. Mendoza N. B. Simon P. D. Cunanan A. L. P. Dizon J. I. W. T. Tarroja M. C. H.. (2020). Coronavirus pandemic anxiety scale (CPAS-11): development and initial validation. Curr. Psychol. 1–9. 10.1007/s12144-020-01193-2 33223781 PMC7664585

[B14] Berthelot N. Lemieux R. Garon-Bissonnette J. Drouin-Maziade C. Martel É. Maziade M. (2020). Uptrend in distress and psychiatric symptomatology in pregnant women during the coronavirus disease 2019 pandemic. Acta Obstet. Gynecol. Scand. 99, 848–855. 10.1111/aogs.13925 32449178

[B15] Black M. M. Walker S. P. Fernald L. C. Andersen C. T. DiGirolamo A. M. Lu C.. (2017). Early childhood development coming of age: science through the life course. Lancet 10064, 77–90. 10.1016/S0140-6736(16)31389-7 27717614 PMC5884058

[B16] Bogavac I. Jeličić L. Nenadović V. Subotić M. Janjić V. (2021). The speech and language profile of a child with turner syndrome– a case study. Clin Linguist Phon. 1–14. 10.1080/02699206.2021.1953610 34309455

[B17] Britto P. R. Lye S. J. Proulx K. Yousafzai A. K. Matthews S. G. Vaivada T.. (2017). Nurturing care: promoting early childhood development. Lancet 10064, 91–102. 10.1016/S0140-6736(16)31390-3 27717615

[B18] Brooks-Gunn J. Han W.-J. Waldfogel J. (2002). Maternal employment and child cognitive outcomes in the first three years of life: the NICHD study of early child care. Child Dev. 73, 1052–1072. 10.1111/1467-8624.00457 12146733

[B19] Bruwer B. Emsley R. Kidd M. Lochner C. Seedat S. (2008). Psychometric properties of the multidimensional scale of perceived social support in youth. Compr. Psychiatry 49, 195–201. 10.1016/j.comppsych.2007.09.002 18243894

[B20] Candelori C. Trumello C. Babore A. Keren M. Romanelli R. (2015). The experience of premature birth for fathers: the application of the clinical interview for parents of high-risk infants (CLIP) to an Italian sample. Front. Psychol. 6:1444. 10.3389/fpsyg.2015.01444 26483712 PMC4586417

[B21] Ceulemans M. Hompes T. Foulon V. (2020). Mental health status of pregnant and breastfeeding women during the COVID-19 pandemic: a call for action. Int. J. Gynecol. Obstetr. 151, 146–147. 10.1002/ijgo.13295 32620037

[B22] Chittleborough C. R. Lawlor D. A. Lynch J. W. (2011). Young maternal age and poor child development: predictive validity from a birth cohort. Pediatrics 127, e1436–e1444. 10.1542/peds.2010-3222 21536608

[B23] Coibion O. Gorodnichenko Y. Weber M. (2020). Labor Markets During the COVID-19 Crisis: A Preliminary View (No. w27017). Cambridge: National Bureau of Economic Research. 10.3386/w27017

[B24] Collett B. R. Wallace E. R. Kartin D. Speltz M. L. (2019). Infant/toddler motor skills as predictors of cognition and language in children with and without positional skull deformation. Childs. Nerv. Syst. 35, 157–163. 10.1007/s00381-018-3986-4 30377774 PMC6447299

[B25] Comaskey B. Roos N. P. Brownell M. Enns M. W. Chateau D. Ruth C. A.. (2017). Maternal depression and anxiety disorders (MDAD) and child development: a manitoba population-based study. PLoS ONE 12:e0177065. 10.1371/journal.pone.0177065 28542256 PMC5443487

[B26] Corbett G. A. Milne S. J. Hehir M. P. Lindow S. W. O'connell M. P. (2020). Health anxiety and behavioural changes of pregnant women during the COVID-19 pandemic. Eur. J. Obstet. Gynecol. 249, 96–97. 10.1016/j.ejogrb.2020.04.022 32317197 PMC7194619

[B27] Cucinotta D. Vanelli M. (2020). WHO declares COVID-19 a pandemic. Acta Biomed. 91:157. 10.23750/abm.v91i1.9397 32191675 PMC7569573

[B28] Dagklis T. Tsakiridis I. Mamopoulos A. Athanasiadis A. Papazisis G. (2020). Anxiety During Pregnancy in the Era of the COVID-19 Pandemic. 10.2139/ssrn.358854234645943

[B29] Dambi J. M. Corten L. Chiwaridzo M. Jack H. Mlambo T. Jelsma J. (2018). A systematic review of the psychometric properties of the cross-cultural translations and adaptations of the multidimensional perceived social support scale (MSPSS). Health Qual. Life Outcomes 16, 1–19. 10.1186/s12955-018-0912-0 29716589 PMC5930820

[B30] Davenport M. H. Meyer S. Meah V. L. Strynadka M. C. Khurana R. (2020). Moms are not OK: COVID-19 and maternal mental health. Front. Glob. Womens Health 1:1. 10.3389/fgwh.2020.00001 34816146 PMC8593957

[B31] Denis A. Callahan S. Bouvard M. (2015). Evaluation of the French version of the multidimensional scale of perceived social support during the postpartum period. Matern. Child Health J. 19, 1245–1251. 10.1007/s10995-014-1630-9 25366102

[B32] Dour H. J. Wiley J. F. Roy-Byrne P. Stein M. B. Sullivan G. Sherbourne C. D.. (2014). Perceived social support mediates anxiety and depressive symptom changes following primary care intervention. Depress. Anxiety 31, 436–442. 10.1002/da.22216 24338947 PMC4136523

[B33] Duncan G. J. Lee K. T. H. Rosales-Rueda M. Kalil A. (2018). Maternal age and child development. Demography 55, 2229–2255. 10.1007/s13524-018-0730-3 30387046 PMC6392079

[B34] Dunkel Schetter C. Tanner L. (2012). Anxiety, depression and stress in pregnancy: implications for mothers, children, research, and practice. Curr. Opin. Psychiatry 25, 141–148. 10.1097/YCO.0b013e3283503680 22262028 PMC4447112

[B35] Durankuş F. Aksu E. (2020). Effects of the COVID-19 pandemic on anxiety and depressive symptoms in pregnant women: a preliminary study. J. Matern. Fetal Neonatal Med. 18, 1–7. 10.1080/14767058.2020.1763946 32419558

[B36] Easter A. Solmi F. Bye A. Taborelli E. Corfield F. Schmidt U.. (2015). Antenatal and postnatal psychopathology among women with current and past eating disorders: longitudinal patterns. Eur. Eat. Disord. Rev. 23, 19–27. 10.1002/erv.2328 25345371 PMC4309475

[B37] Falster K. Hanly M. Banks E. Lynch J. Chambers G. Brownell M.. (2018). Maternal age and offspring developmental vulnerability at age five: a population-based cohort study of Australian children. PLoS Med. 15:e1002558. 10.1371/journal.pmed.1002558 29689098 PMC5915778

[B38] Fan S. Guan J. Cao L. Wang M. Zhao H. Chen L.. (2021). Psychological effects caused by COVID-19 pandemic on pregnant women: a systematic review with meta-analysis. Asian J. Psychiatry 56:102533. 10.1016/j.ajp.2020.102533 33418283 PMC7833174

[B39] Flannery R. B. Jr. Wieman D. (1989). Social support, life stress, and psychological distress: an empirical assessment. J. Clin. Psychol. 45, 867–872. 10.1002/1097-4679(198911)45:6<867::AID-JCLP2270450606>3.0.CO;2-I 2613895

[B40] Floris L. Irion O. Courvoisier D. (2017). Influence of obstetrical events on satisfaction and anxiety during childbirth: a prospective longitudinal study. Psychol. Health Med. 22, 969–977. 10.1080/13548506.2016.1258480 27855515

[B41] Glasheen C. Richardson G. A. Fabio A. (2010). A systematic review of the effects of postnatal maternal anxiety on children. Arch. Womens. Ment. Health 13, 61–74. 10.1007/s00737-009-0109-y 19789953 PMC3100191

[B42] Grant K. A. McMahon C. Austin M. P. (2008). Maternal anxiety during the transition to parenthood: a prospective study. J. Affect. Disord. 108, 101–111. 10.1016/j.jad.2007.10.002 18001841

[B43] Gunning M. D. Denison F. C. Stockley C. J. Ho S. P. Sandhu H. K. Reynolds R. M. (2010). Assessing maternal anxiety in pregnancy with the state-trait anxiety inventory (STAI): issues of validity, location and participation. J. Reprod. Infant Psychol. 28, 266–273. 10.1080/02646830903487300

[B44] Hamid F. Asif A. Haider I. I. (2008). Study of anxiety and depression during pregnancy. Pak. J. Med. Sci. 24, 861–4.

[B45] Hashim M. Coussa A. Al Dhaheri A. S. Al Marzouqi A. Cheaib S. Salame A.. (2021). Impact of coronavirus 2019 on mental health and lifestyle adaptations of pregnant women in the United Arab Emirates: a cross-sectional study. BMC Pregn. Childbirth. 21:515. 10.1186/s12884-021-03941-z 34281501 PMC8287543

[B46] Hernández-Martínez C. Arija V. Balaguer A. Cavallé P. Canals J. (2008). Do the emotional states of pregnant women affect neonatal behaviour? Early Hum. Dev. 84, 745–750. 10.1016/j.earlhumdev.2008.05.002 18571345

[B47] Hessami K. Romanelli C. Chiurazzi M. Cozzolino M. (2020). COVID-19 pandemic and maternal mental health: a systematic review and meta-analysis. J. Mater. Fetal Neonatal Med. 1–8. 10.1080/14767058.2020.1843155 33135523

[B48] Hetkamp M. Schweda A. Bäuerle A. Weismüller B. Kohler H. Musche V.. (2020). Sleep disturbances, fear, and generalized anxiety during the COVID-19 shut down phase in Germany: relation to infection rates, deaths, and German stock index DAX. Sleep Med. 75, 350–353. 10.1016/j.sleep.2020.08.033 32950879 PMC7470793

[B49] Huizink A. C. Menting B. Oosterman M. Verhage M. L. Kunseler F. C. Schuengel C. (2014). The interrelationship between pregnancy-specific anxiety and general anxiety across pregnancy: a longitudinal study. J. Psychosom. Obstet. Gynaecol. 35, 92–100. 10.3109/0167482X.2014.944498 25093467

[B50] Iverson J. M. (2010). Developing language in a developing body: the relationship between motor development and language development. J. Child Lang. 37:229. 10.1017/S0305000909990432 20096145 PMC2833284

[B51] Johnson K. (2013). Maternal-Infant bonding: a review of literature. Int. J. Childbirth Educ. 28, 17–22.

[B52] Julian L. J. (2011). Measures of anxiety: state-trait anxiety inventory (STAI), beck anxiety inventory (BAI), and hospital anxiety and depression scale-anxiety (HADS-A). Arthritis Care Res. 63 (Suppl. 11), S467–S472. 10.1002/acr.20561 22588767 PMC3879951

[B53] Khoury J. E. Atkinson L. Bennett T. Jack S. M. Gonzalez A. (2021). COVID-19 and mental health during pregnancy: the importance of cognitive appraisal and social support. J. Affect. Disord. 282, 1161–1169. 10.1016/j.jad.2021.01.027 33601691 PMC7837227

[B54] Kikkert H. K. Middelburg K. J. Hadders-Algra M. (2010). Maternal anxiety is related to infant neurological condition, paternal anxiety is not. Early Hum. Dev. 86, 171–177. 10.1016/j.earlhumdev.2010.02.004 20226603

[B55] Killgore W. D. Cloonan S. A. Taylor E. C. Dailey N. S. (2021). Mental health during the first weeks of the COVID-19 pandemic in the United States. Front. Psychiatry. 12:535. 10.3389/fpsyt.2021.561898 33967841 PMC8100184

[B56] King S. Dancause K. Turcotte-Tremblay A. M. Veru F. Laplante D. P. (2012). Using natural disasters to study the effects of prenatal maternal stress on child health and development. Birth Defects Res. C Embryo Today 96, 273–288. 10.1002/bdrc.21026 24203917

[B57] Kotabagi P. Fortune L. Essien S. Nauta M. Yoong W. (2020). Anxiety and depression levels among pregnant women with COVID-19. Acta Obstet. Gynecol. Scand. 99, 953–954. 10.1111/aogs.13928 32474914 PMC7300632

[B58] Le Bas G. Youssef G. Macdonald J. A. Teague S. Mattick R. Honan I.. (2021). The role of antenatal and postnatal maternal bonding in infant development. J. Am. Acad. Child Adolesc. Psychiatry 17. 10.1016/j.jaac.2021.08.024 34555489

[B59] Leach L. S. Poyser C. Fairweather-Schmidt K. (2017). Maternal perinatal anxiety: a review of prevalence and correlates. Clin Psychol. 21, 4–19. 10.1111/cp.1205816421038

[B60] Lebel C. MacKinnon A. Bagshawe M. Tomfohr-Madsen L. Giesbrecht G. (2020). Elevated depression and anxiety symptoms among pregnant individuals during the COVID-19 pandemic. J. Affect. Disord. 277, 5–13. 10.1016/j.jad.2020.07.126 32777604 PMC7395614

[B61] Leonard H. C. Bedford R. Pickles A. Hill E. L. BASIS Team. (2015). Predicting the rate of language development from early motor skills in at-risk infants who develop autism spectrum disorder. Res. Autism Spectr. Disord. 13, 15–24. 10.1016/j.rasd.2014.12.012 31410102 PMC6692163

[B62] Leonard H. C. Hill E. L. (2014). The impact of motor development on typical and atypical social cognition and language: a systematic review. Child Adolesc. Ment. Health 19, 163–170. 10.1111/camh.12055 32878369

[B63] Libertus K. Hauf P. (2017). Motor skills and their foundational role for perceptual, social, and cognitive development. Front. Psychol. 8:301. 10.3389/fpsyg.2017.00301 28321199 PMC5337521

[B64] Libertus K. Violi D. A. (2016). Sit to talk: relation between motor skills and language development in infancy. Front. Psychol. 7:475. 10.3389/fpsyg.2016.00475 27065934 PMC4815289

[B65] Liu C. H. Erdei C. Mittal L. (2021). Risk factors for depression, anxiety, and PTSD symptoms in perinatal women during the COVID-19 pandemic. Psychiatry Res. 295:113552. 10.1016/j.psychres.2020.113552 33229122 PMC7904099

[B66] Luby J. L. (2015). Poverty's most insidious damage: the developing brain. JAMA Pediatr. 169, 810–811. 10.1001/jamapediatrics.2015.1682 26191940

[B67] Mappa I. Distefano F. A. Rizzo G. (2020). Effects of coronavirus 19 pandemic on maternal anxiety during pregnancy: a prospectic observational study. J. Perinat. Med. 48, 545–550. 10.1515/jpm-2020-0182 32598320

[B68] Mappa I. Luviso M. Distefano F. A. Carbone L. Maruotti G. M. Rizzo G. (2021). Women perception of SARS-CoV-2 vaccination during pregnancy and subsequent maternal anxiety: a prospective observational study. J. Matern. Fetal Neonatal Med. 1–4. 10.1080/14767058.2021.1910672 33843419

[B69] Martini J. Petzoldt J. Einsle F. Beesdo-Baum K. Höfler M. Wittchen H. U. (2015). Risk factors and course patterns of anxiety and depressive disorders during pregnancy and after delivery: a prospective-longitudinal study. J. Affect. Disord. 175, 385–395. 10.1016/j.jad.2015.01.012 25678171

[B70] Meyerowitz-Katz G. Bhatt S. Ratmann O. Brauner J. M. Flaxman S. Mishra S.. (2021). Is the cure really worse than the disease? The health impacts of lockdowns during COVID-19. BMJ Global Health 6:e006653. 10.1136/bmjgh-2021-006653 34281914 PMC8292804

[B71] Motrico E. Bina R. Domínguez-Salas S. Mateus V. Contreras-García Y. Carrasco-Portiño M.. (2021). Impact of the Covid-19 pandemic on perinatal mental health (Riseup-PPD-COVID-19): protocol for an international prospective cohort study. BMC Public Health 21:368. 10.1186/s12889-021-10330-w 33596889 PMC7887558

[B72] Moyer C. A. Compton S. D. Kaselitz E. Muzik M. (2020). Pregnancy-related anxiety during COVID-19: a nationwide survey of 2740 pregnant women. Arch. Womens Ment. Health 23, 757–765. 10.1007/s00737-020-01073-5 32989598 PMC7522009

[B73] Naveed S. Lashari U. G. Waqas A. Bhuiyan M. Meraj H. (2018). Gender of children and social provisions as predictors of unplanned pregnancies in Pakistan: a cross-sectional survey. BMC Res. Notes 11:587. 10.1186/s13104-018-3696-8 30107823 PMC6092811

[B74] Nip I. S. Green J. R. Marx D. B. (2011). The co-emergence of cognition, language, and speech motor control in early development: a longitudinal correlation study. J. Commun. Disord. 44, 149–160. 10.1016/j.jcomdis.2010.08.002 21035125 PMC3057350

[B75] Nomura R. Tavares I. Ubinha A. C. Costa M. L. Opperman M. L. Brock M.. (2021). Impact of the COVID-19 pandemic on MATERNAL ANXIETY In Brazil. J. Clin. Med. 10:620. 10.3390/jcm10040620 33562012 PMC7914962

[B76] Nowacka U. Kozlowski S. Januszewski M. Sierdzinski J. Jakimiuk A. Issat T. (2021). COVID-19 pandemic-related anxiety in pregnant women. Int. J. Environ. Res. Public Health 18:7221. 10.3390/ijerph18147221 34299673 PMC8307177

[B77] O'Connor T. G. Heron J. Golding J. Beveridge M. Glover V. (2002). Maternal antenatal anxiety and children's behavioural/emotional problems at 4 years: report from the avon longitudinal study of parents and children. Br. J. Psychiatry 180, 502–508. 10.1192/bjp.180.6.502 12042228

[B78] O'Connor T. G. Monk C. Fitelson E. M. (2014). Practitioner review: maternal mood in pregnancy and child development–implications for child psychology and psychiatry. J. Child Psychol. Psychiatry. 55, 99–111. 10.1111/jcpp.12153 24127722 PMC3982916

[B79] Özpelit M. E. Özpelit E. Dogan N. B. Pekel N. Ozyurtlu F. Yilmaz A.. (2015). Impact of anxiety level on circadian rhythm of blood pressure in hypertensive patients. Int. J. Clin. Exp. Med. 8, 16252–16258. 26629141 PMC4659029

[B80] Papadopoulou C. Kotronoulas G. Schneider A. Miller M. I. McBride J. Polly Z.. (2017). Patient-reported self-efficacy, anxiety, and health-related quality of life during chemotherapy: results from a longitudinal study. Oncol. Nurs. Forum 44, 127–136. 10.1188/17.ONF.127-136 27991612

[B81] Paredes M. R. Apaolaza V. Fernandez-Robin C. Hartmann P. Yañez-Martinez D. (2021). The impact of the COVID-19 pandemic on subjective mental well-being: the interplay of perceived threat, future anxiety and resilience. Pers. Individ. Differ. 170:110455. 10.1016/j.paid.2020.110455 33071413 PMC7552984

[B82] Pejičić M. Ristić M. Andelković V. (2018). The mediating effect of cognitive emotion regulation strategies in the relationship between perceived social support and resilience in postwar youth. J. Community Psychol. 46, 457–472. 10.1002/jcop.2195125855820

[B83] Piek J. P. Dawson L. Smith L. M. Gasson N. (2008). The role of early fine and gross motor development on later motor and cognitive ability. Hum. Mov. Sci. 27, 668–681. 10.1016/j.humov.2007.11.002 18242747

[B84] Quinlivan J. Lambregtse-van den Berg M. (2020). Will COVID-19 impact upon pregnancy, childhood and adult outcomes? A call to establish national longitudinal datasets. J. Psychosom. Obstet. Gynaecol. 41, 165–166. 10.1080/0167482X.2020.1775925 32838631

[B85] Rakonjac M. Cuturilo G. Stevanovic M. Jelicic L. Subotic M. Jovanovic I.. (2016). Differences in speech and language abilities between children with 22q11. 2 deletion syndrome and children with phenotypic features of 22q11. 2 deletion syndrome but without microdeletion. Res. Dev. Disabil. 55, 322–329. 10.1016/j.ridd.2016.05.006 27235769

[B86] Rees S. Channon S. Waters C. S. (2019). The impact of maternal prenatal and postnatal anxiety on children's emotional problems: a systematic review. Eur. Child Adolesc. Psychiatry 28, 257–280. 10.1007/s00787-018-1173-5 29948234 PMC6510846

[B87] Ren X. Huang W. Pan H. Huang T. Wang X. Ma Y. (2020). Mental health during the Covid-19 outbreak in China: a meta-analysis. Psychiatr. Q. 91, 1033–45. 10.1007/s11126-020-09796-5 32642822 PMC7343383

[B88] Rothenberger S. E. Moehler E. Reck C. Resch F. (2011). Prenatal stress: course and interrelation of emotional and physiological stress measures. Psychopathology 44, 60–67. 10.1159/000319309 21072001

[B89] Ryan J. Mansell T. Fransquet P. Saffery R. (2017). Does maternal mental well-being in pregnancy impact the early human epigenome? Epigenomics 9, 313–332. 10.2217/epi-2016-0118 28140666

[B90] Saarni C. Campos J. J. Camras L. A. Witherington D. (2006). “Emotional development: action, communication, and understanding,” in Handbook of Child Psychology: Social, Emotional, and Personality Development, eds N. Eisenberg, W. Damon, and R. M. Lerner (Hoboken, New Jersey: John Wiley & Sons, Inc), 226–299. 10.1002/9780470147658.chpsy0305

[B91] Saccone G. Florio A. Aiello F. Venturella R. De Angelis M. C. Locci M.. (2020). Psychological impact of coronavirus disease 2019 in pregnant women. Am. J. Obstet. Gynecol. 223, 293–295. 10.1016/j.ajog.2020.05.003 32387321 PMC7204688

[B92] Sameroff A. (2009). The Transactional Model of Development: How Children and Contexts Shape Each Other. New York, NY: Wiley. 10.1037/11877-000

[B93] Sharif M. Ahmed Zaidi A. W. Malik A. Hagaman A. Maselko J. LeMasters K.. (2021). Psychometric validation of the Multidimensional scale of perceived social support during pregnancy in rural Pakistan. Front. Psychol. 12:2202. 10.3389/fpsyg.2021.601563 34211414 PMC8239233

[B94] Shigemura J. Ursano R. J. Morganstein J. C. Kurosawa M. Benedek D. M. (2020). Public responses to the novel 2019 coronavirus (2019-nCoV) in Japan: mental health consequences and target populations. Psychiatry Clin. Neurosci. 74, 281–282. 10.1111/pcn.12988 32034840 PMC7168047

[B95] Skoda E. M. Bäuerle A. Schweda A. Dörrie N. Musche V. Hetkamp M.. (2020). Severely increased generalized anxiety, but not COVID-19-related fear in individuals with mental illnesses: a population based cross-sectional study in Germany. Int. J. Soc. Psychiatry 67, 550–558. 10.1177/0020764020960773 33040668

[B96] Spielberger C. D. Gorsuch R. L. Lushene R. Vagg P. R. Jacobs G. A. (1983). State-Trait Anxiety Inventory for Adults. Palo Alto: Consulting Psychologists Press Inc. 10.1037/t06496-000

[B97] Spielberger C. D. Gorsuch R. L. Lushene R. Vagg P. R. Jacobs G. A. (2000). Priručnik za Upitnik Anksioznosti Kao Stanja i Osobine Ličnosti [State-Trait Anxiety Inventory (Form Y)]. Jastrebarsko: Naklada Slap.

[B98] Stašević-Karličić I. ordević V. Stašević M. Subotić T. Filipović Z. Ignjatović-Ristić. (2020). Perspectives on mental health services during the COVID-19 epidemic in Serbia. Srp Arh. Celok Lek. 148, 379–382. 10.2298/SARH200504028S

[B99] Subota N. (2003). Child's Drawing—Speech and Language Development and Cognitive Functioning. Belgrade: Zaduzbina Andrejevic.

[B100] Sun F. Zhu J. Tao H. Ma Y. Jin W. (2021). A systematic review involving 11,187 participants evaluating the impact of COVID-19 on anxiety and depression in pregnant women. J. Psychosom. Obstetr. Gynecol. 42, 91–99. 10.1080/0167482X.2020.1857360 33327827

[B101] Sut H. K. Kucukkaya B. (2020). Anxiety, depression, and related factors in pregnant women during the COVID-19 pandemic in Turkey: A web-based cross-sectional study. Perspect. Psychiatr. Care. 57, 860–868. 10.1111/ppc.12627 32989798 PMC7537279

[B102] Tearne J. E. (2015). Older maternal age and child behavioral and cognitive outcomes: a review of the literature. Fertil. Steril. 103, 1381–1391. 10.1016/j.fertnstert.2015.04.027 26041693

[B103] Tomašević-Todorovic S. T. Hanna F. Boskovic K. Filipovic D. Vidovic V. Filipovic K. (2012). Motor ability and emotions in rheumatoid arthritis patients. J. Neurol. Neurophysiol. 3, 1–5.11352254

[B104] Tomfohr-Madsen L. M. Racine N. Giesbrecht G. F. Lebel C. Madigan S. (2021). Depression and anxiety in pregnancy during COVID-19: a rapid review and meta-analysis. Psychiatry Res. 300:113912. 10.1016/j.psychres.2021.113912 33836471 PMC9755112

[B105] Usher K. Durkin J. Bhullar N. (2020). The COVID-19 pandemic and mental health impacts. Int. J. Ment. Health Nurs. 29, 315–318. 10.1111/inm.12726 32277578 PMC7262128

[B106] Van den Bergh B. R. Mulder E. J. Mennes M. Glover V. (2005). Antenatal maternal anxiety and stress and the neurobehavioural development of the fetus and child: links and possible mechanisms. A review. Neurosci. Biobehav. Rev. 29, 237–258. 10.1016/j.neubiorev.2004.10.007 15811496

[B107] Vujović M. Sovilj M. Plešinac S. Rakonjac M. Jeličić L. Adamović T.. (2019). Effect of antenatal maternal anxiety on the reactivity of fetal cerebral circulation to auditory stimulation, and early child development. Srp Arh. Celok. Lek. 147, 327–334. 10.2298/SARH181002024V

[B108] Wang C. Pan R. Wan X. Tan Y. Xu L. Ho C. S.. (2020). Immediate psychological responses and associated factors during the initial stage of the 2019 coronavirus disease (COVID-19) epidemic among the general population in China. Int. J. Environ. Res. Public Health 17:1729. 10.3390/ijerph17051729 32155789 PMC7084952

[B109] Wang M. V. Lekhal R. Aarø L. E. Schjolberg S. (2014). Co-occurring development of early childhood communication and motor skills: results from a population-based longitudinal study. Child Care Health Dev. 40, 77–84. 10.1111/cch.12003 22970997

[B110] Wu Y. Zhang C. Liu H. Duan C. Li C. Fan J.. (2020). Perinatal depressive and anxiety symptoms of pregnant women during the coronavirus disease 2019 outbreak in China. Am. J. Obstet. Gynecol. 223, 240.e1–240.e9. 10.1016/j.ajog.2020.05.009 32437665 PMC7211756

[B111] Xiang Y. T. Yang Y. Li W. Zhang L. Zhang Q. Cheung T.. (2020). Timely mental health care for the 2019 novel coronavirus outbreak is urgently needed. Lancet Psychiatry 7, 228–229. 10.1016/S2215-0366(20)30046-8 32032543 PMC7128153

[B112] Yan H. Ding Y. Guo W. (2020). Mental health of pregnant and postpartum women during the coronavirus disease 2019 pandemic: a systematic review and meta-analysis. Front. Psychol. 11:617001. 10.3389/fpsyg.2020.617001 33324308 PMC7723850

[B113] Yue C. Liu C. Wang J. Zhang M. Wu H. Li C.. (2021). Association between social support and anxiety among pregnant women in the third trimester during the coronavirus disease 2019 (COVID-19) epidemic in Qingdao, China: the mediating effect of risk perception. Int. J. Soc. Psychiatry 67, 120–127. 10.1177/0020764020941567 32643510 PMC7348553

[B114] Zhang Y. Ma Z. F. (2020). Psychological responses and lifestyle changes among pregnant women with respect to the early stages of COVID-19 pandemic. Int. J. Soc. Psychiatry 67, 344–350. 10.1177/0020764020952116 32815434 PMC8191160

[B115] Zilver S. J. M. Broekman B. F. P. Hendrix Y. M. G. A. de Leeuw R. A. Mentzel S. V. van Pampus M. G.. (2021). Stress, anxiety and depression in 1466 pregnant women during and before the COVID-19 pandemic: a dutch cohort study. J. Psychosom. Obstetr. Gynecol. 42, 108–114. 10.1080/0167482X.2021.1907338 33900872

[B116] Zimet G. D. Dahlem N. W. Zimet S. G. Farley G. K. (1988). The multidimensional scale of perceived social support. J. Pers. Assess. 52, 30–41. 10.1207/s15327752jpa5201_2 2280326

