# Supplementary material for: The Impact of Maternal Anxiety on Early Child Development During the COVID-19 Pandemic
Source: Front Psychol. 2021 Dec 22;12:792053. doi: 10.3389/fpsyg.2021.792053 (PMC8728063; doi:10.3389/fpsyg.2021.792053)
Supplement: Supplementary file 1 [file Table_1.docx]

| **APPENDIX A** I Explanation of new grouping variables of interaction terms used in One–Way ANOVA statistical test: a) Employment_COVID-19 related fear,  b) Employment_STAI-T level, c) Number of children_COVID-19 related fear_STAI-T level, d) Employment_COVID-19 related fear_STAI-T level | |
| --- | --- |
| a) Explanation of Employment_COVID-19 related fear grouping variable values   \| Employment \| COVID-19 related fear \| Grouping value \| \| --- \| --- \| --- \| \| Yes \| No \| 1 \| \| Sometimes \| 2 \| \| Yes \| 3 \| \| No \| No \| 4 \| \| Sometimes \| 5 \| \| Yes \| 6 \| | b) Explanation of Employment _STAI-T level grouping variable values   \| STAI-T level \| Employment \| Grouping value \| \| --- \| --- \| --- \| \| Intermediate \| Yes \| 1 \| \| No \| 2 \| \| High \| Yes \| 3 \| \| No \| 4 \| |
| c) Explanation of Number of children_COVID-19 related fear_STAI-T level grouping variable values   \| STAI-T level \| Number of  children \| COVID-19 related fear \| Grouping value \| \| --- \| --- \| --- \| --- \| \| Intermediate \| One \| No \| 1 \| \| Sometimes \| 2 \| \| Yes \| 3 \| \| Two or more \| No \| 4 \| \| Sometimes \| 5 \| \| Yes \| 6 \| \| High \| One \| No \| 7 \| \| Sometimes \| 8 \| \| Yes \| 9 \| \| Two or more \| No \| 10 \| \| Sometimes \| 11 \| \| Yes \| 12 \| | d) Explanation of Employment_COVID-19 related fear_STAI-T level grouping variable values   \| STAI-T level \| Employment \| COVID-19 related fear \| Grouping value \| \| --- \| --- \| --- \| --- \| \| Intermediate \| Yes \| No \| 1 \| \| Sometimes \| 2 \| \| Yes \| 3 \| \| No \| No \| 4 \| \| Sometimes \| 5 \| \| Yes \| 6 \| \| High \| Yes \| No \| 7 \| \| Sometimes \| 8 \| \| Yes \| 9 \| \| No \| No \| 10 \| \| Sometimes \| 11 \| \| Yes \| 12 \| |
