# Supplementary material for: The Impact of Maternal Anxiety on Early Child Development During the COVID-19 Pandemic
Source: Front Psychol. 2021 Dec 22;12:792053. doi: 10.3389/fpsyg.2021.792053 (PMC8728063; doi:10.3389/fpsyg.2021.792053)
Supplement: Supplementary file 2 [file Table_2.docx]

**APPENDIX B** I Tests of Between-Subjects Effects for dependent variables SPEECH-LANGUAGE, MOTOR skills, COGNITION and SOCIO-EMOTIONAL achievement. Only statistically significant factors and interactions are presented

| Source | F | Sig. | Partial η^2^ | Observed Power |
| --- | --- | --- | --- | --- |
| SPEECH-LANGUAGE achievement | | | | |
| Corrected Model | 3.902 | .000 | .406 | 1.00 |
| Maternal Age | 5.101 | .026 | .041 | .610 |
| Employed | 8.146 | .005 | .064 | .808 |
| Employed * COVID-19 related fear | 6.039 | .003 | .091 | .877 |
| Employed * STAI-T level | 8.115 | .005 | .063 | .807 |
| COVID-19 related fear * STAI-T level | 11.332 | .000 | .159 | .992 |
| Number of children * COVID-19 related fear * STAI-T level | 26.626 | .000 | .182 | .999 |
| MOTOR skills achievement | | | | |
| Corrected Model | 3.031 | .000 | .347 | .999 |
| COVID-19 related fear | 4.357 | .015 | .068 | .745 |
| STAI-T level | 4.243 | .042 | .034 | .533 |
| Number of children * STAI-T level | 5.402 | .022 | .043 | .635 |
| COVID-19 related fear * STAI-T level | 5.742 | .004 | .087 | .859 |
| Employed * COVID-19 related fear * STAI-T level | 11.823 | .001 | .090 | .927 |
| Number of children * COVID-19 related fear * STAI-T level | 6.896 | .010 | .054 | .741 |
| COGNITION achievement | | | | |
| Corrected Model | 2.555 | .001 | .309 | .997 |
| Employed * COVID-19 related fear | 5.641 | .005 | .086 | .853 |
| COVID-19 related fear * STAI-T level | 8.329 | .000 | .122 | .959 |
| Number of children * COVID-19 related fear * STAI-T level | 9.139 | .003 | .071 | .851 |
| SOCIO-EMOTIONAL achievement | | | | |
| Corrected Model | 3.673 | .000 | .391 | 1.000 |
| Employed | 14.166 | .000 | .106 | .962 |
| Employed * COVID-19 related fear | 4.125 | .019 | .064 | .720 |
| Employed * STAI-T level | 17.443 | .000 | .127 | .985 |
| COVID-19 related fear * STAI-T level | 5.962 | .003 | .090 | .873 |
| Number of children * COVID-19 related fear * STAI-T level | 22.924 | .000 | .160 | .997 |
